# Agriculture

**Published:** 1880-06

**Authors:** 


### AGRICULTURE

Pursued with intelligent industry, affords a larger number of high advantages than any other occupation of human life; it strengthens the body, invigorates the mind; and while it refines the sentiments, it purifies the heart, by compelling it to look upward for reliance and help towards Him *who giveth rain and fruitful seasons.* It curbs inordinate ambitions, by yielding a moderate remuneration for toil, while at the same time it imparts a feeling of quiet confidence in the future, from the declaration that while the world stands, *seed time and harvest shall not cease.* The young man brought up to till the soil, begins to feel gradually that the rewards of his toil, are proportioned to his labor, and this imparts by degrees a spirit of self-reliance, which begets independence, and an amount of industrious activities, worth more to that young man, in his after conflicts with the world, than the inheritance of unearned thousands.

